# Improving Robustness of High-Low-Order Coupled Networks against Malicious Attacks Based on a Simulated Annealing Algorithm

**DOI:** 10.3390/e26010008

**Published:** 2023-12-21

**Authors:** Chengjun Zhang, Yifan Xie, Yadang Chen, Wenbin Yu, Gaofeng Xiang, Peijun Zhao, Yi Lei

**Affiliations:** 1 School of Computer Science, Nanjing University of Information Science and Technology, Nanjing 210044, China; 2 Wuxi Institute of Technology, Nanjing University of lnformation Science & Technology, Wuxi 214000, China; 3 School of Software, Nanjing University of Information Science and Technology, Nanjing 210044, China; 4 Jiangsu Collaborative Innovation Center of Atmospheric Environment and Equipment Technology (CI-CAEET), Nanjing University of Information Science and Technology, Nanjing 210044, China

**Keywords:** malicious attacks, coupled networks, simulated annealing algorithm, robustness

## Abstract

Malicious attacks can cause significant damage to the structure and functionality of complex networks. Previous research has pointed out that the ability of networks to withstand malicious attacks becomes weaker when networks are coupled. However, traditional research on improving the robustness of networks has focused on individual low-order or higher-order networks, lacking studies on coupled networks with higher-order and low-order networks. This paper proposes a method for optimizing the robustness of coupled networks with higher-order and low-order based on a simulated annealing algorithm to address this issue. Without altering the network’s degree distribution, the method rewires the edges, taking the robustness of low-order and higher-order networks as joint optimization objectives. Making minimal changes to the network, the method effectively enhances the robustness of coupled networks. Experiments were conducted on Erdős–Rényi random networks (ER), scale-free networks (BA), and small-world networks (SW). Finally, validation was performed on various real networks. The results indicate that this method can effectively enhance the robustness of coupled networks with higher-order and low-order.

## 1. Introduction

With the progressive advancement of human civilization, many intricate systems, including communication systems, transportation systems, and the Internet, have witnessed remarkable expansion. These complex systems exhibit shared characteristics of self-organization, adaptability, and evolution. In recent decades, researchers have begun employing network science methodologies to explore the intricacies of complex systems. Propelled by advancements in network information technology, notably exemplified by the Internet, the proliferation of complex networks has gained momentum since the 1980s [1,2,3]. Researchers have harnessed graph theory as a foundational framework to investigate complex networks’ properties, leveraging high-performance computers to simulate network dynamics [4,5]. Complex networks have become interwoven with human production and daily existence in the present-day milieu. Communication, transportation, social, and biological networks profoundly influence human activities [6,7]. Consequently, scholars from diverse disciplines are ardently engaged in studying complex networks.

In the early 21st century, Milo et al. introduced network motifs to elucidate the underlying structural principles governing complex networks. Network motifs pertain to recurring patterns of interconnections within complex networks that exceed the frequency observed in random networks. These patterns have been observed in diverse domains, such as ecological food webs, neural networks, and the World Wide Web [8]. Shen-Orr et al. investigated network motifs within the transcriptional interaction network of Escherichia coli. Their study unveiled that most of the network’s architecture comprised a small yet crucial set of three motifs that exhibited repetitive occurrences. Notably, each motif manifested specific functional characteristics related to distinct facets of gene expression. The research methodologies employed in this network analysis also hold promise for elucidating other biological networks [9]. The study of network motifs mentioned above has promoted the development of complex networks and laid the foundation for studying higher-order networks.

Network robustness has always been an important issue. The functionality of a network is often contingent upon the integrity of its giant components, and any significant compromise to these giant components can consequently impact the overall functionality of the network [10]. The study of complex network robustness holds profound implications for real-world systems. In the infrastructure domain, the study of network robustness involves assessment of the stability of infrastructure network designs, thereby facilitating the development of more robust infrastructure networks [11]. In economics, the robustness of banking network systems is crucial in reducing latent financial risks within economic systems [12]. Furthermore, it has been observed that the robustness of higher-order networks plays a pivotal role in various complex networks. Instances include the significant influence of higher-order structures in social and neural networks, such as triangular patterns and bi-directional wedges, respectively [13,14]. Moreover, many real-world networks are interdependent and engage in mutual interactions. Addressing the enhancement of network robustness in scenarios where low-order and high-order networks interact, particularly in the aftermath of deliberate attacks causing damage to the network, remains a significant research question.

This article presents an approach to optimize the robustness of high-low-order coupled networks by utilizing a simulated annealing algorithm. In complex networks, there are many problems involving optimization. Problems in complex networks usually have many locally optimal solutions, which greedy algorithms can easily fall into. We thus need to use more effective algorithms to address the local optimum issue, such as the simulated annealing algorithm, the ant colony algorithm, and the genetic algorithm. In this paper, we chose the relatively simple and effective simulated annealing algorithm. The experiments show that our algorithm performs well. By preserving the original (low-order) network degree distribution, this method effectively enhances the structural arrangement of both the low-order and higher-order networks by strategically rewiring the low-order networks’ interconnections. The objective is to fortify the robustness of both low-order and higher-order networks, thereby bolstering the overall robustness of the high-low-order coupled network.

## 2. Related Works

Traditional research on the robustness of complex networks typically focuses on low-order networks. Albert et al. found that many scale-free networks exhibit strong robustness, meaning that even if a portion of the nodes in the network fails, this rarely leads to the loss of overall functionality. This robustness is attributed to redundant connections in complex networks [15]. Herrmann et al. used a Monte Carlo method to swap edges in a network while preserving the degree distribution to enhance network robustness. The experimental results showed significant effectiveness, and the generated networks exhibited a structure resembling an onion shape [16]. Smolyak et al. proposed a method to protect critical nodes from mitigating cascading failures and validated the effectiveness of this method on financial networks [17]. Lin W et al. proposed a novel network attack technique based on a genetic algorithm that can operate in linear time for the size of the network, and the results showed that the method struck a balance between attack quality and computational complexity [18]. Zhou B et al. found that malicious attackers with jamming capabilities can exploit the vulnerability of the k-core structure to attack the network, emphasizing the potential vulnerability of the k-core structure and the need to pay attention to its robustness to ensure the security of graph algorithms [19].

Over the years, researchers have explored the impact of network motifs on complex networks. It was not until 2016 that Benson et al. discovered that complex networks could exhibit prosperous higher-order organization through different network motifs. Battiston et al. argued that higher-order structures are better for depicting the structural organization of many social networks, biological networks, and other complex networks. In reality, complex networks often involve higher-order interactions among three or more units, while network representations inherently describe only pairwise interactions. They proposed that higher-order interactions can give rise to collective behavior and described three critical challenges faced by the higher-order physics of systems [20]. Xia et al., based on percolation theory, deliberately and randomly attacked networks by progressively removing nodes or edges to analyze the robustness of both low-order and higher-order networks. The results showed that higher-order networks tend to be more fragile than low-order networks [21]. Lai Y et al. investigated the robustness of interdependent higher-order networks by performing random attacks. The robustness of the interdependent higher-order network structure was found to be higher than the original interdependent network structure [22].

However, the studies mentioned above have predominantly focused on investigating the characteristics of either low-order or higher-order networks in isolation, overlooking the impact of their mutual interactions on the network. Research has shown that disrupting the connectivity of higher-order networks can significantly impair the functionality of low-order networks. Additionally, the functionality of complex networks depends on the presence of giant components in the low-order networks, indicating that network functionality is influenced by both the low-order network itself and its corresponding higher-order network. The rapid development of complexity science has deepened our understanding of complex networks, and research on higher-order networks, coupled networks, and network robustness has played a vital role in exploring the structure and functionality of complex networks. However, there are still gaps in current research, particularly regarding the cascading failures that occur due to the interactions between networks when they experience failures. In the face of network attacks and destruction, enhancing the robustness of low-order and higher-order networks when they interact remains a challenge.

## 3. Methods

### 3.1. Network Motifs and Higher-Order Networks

**Figure 1:**
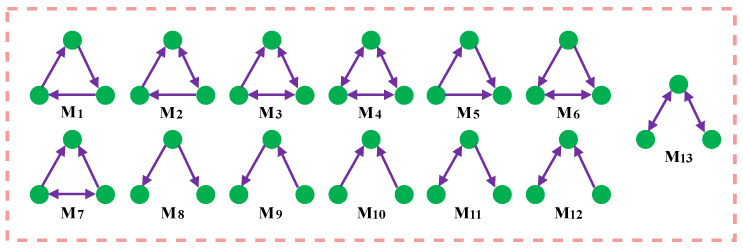
13 types of motifs for directed networks.

Milo et al. revealed the structural principles of complex networks by defining network motifs. Network motifs are network subgraphs composed of three or more nodes, which are fundamental in constructing complex networks and play a crucial role in network functionality [8,23]. Figure 1 presents 13 different three-node network motifs in a directed network. For instance, the feed-forward loop (M5 in Figure 1) is vital in transcriptional regulatory networks and social networks, while the open bidirectional wedge (M13 in Figure 1) is critical in central brain structures.

**Figure 2:**
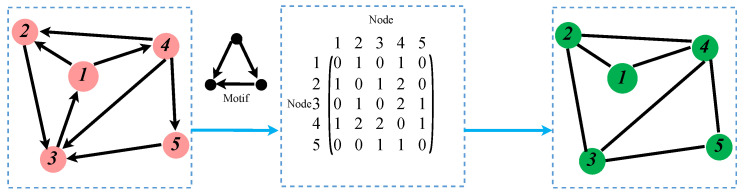
Generating higher-order networks corresponding to low-order networks based on motifs.

Using specific network motifs, corresponding higher-order networks can be generated based on the original network. The specific generation process is illustrated in Figure 2. Given a network and a motif, the adjacency matrix can be generated by computing the number of times two nodes appear together in the motif. Based on this adjacency matrix, an undirected higher-order network can be constructed. Exploring the characteristics of higher-order networks can help researchers identify essential nodes within the network and develop strategies to protect these critical nodes through specialized means [24]. Furthermore, higher-order networks can be employed to study the spread of pollutants in the air, providing valuable insights for environmental governance [25]. Therefore, higher-order networks play a significant role in complex networks, and studying them allows for a deeper understanding of networks’ properties and dynamic behaviors [20,26].

### 3.2. High-Low-Order Coupled Network

**Figure 3:**
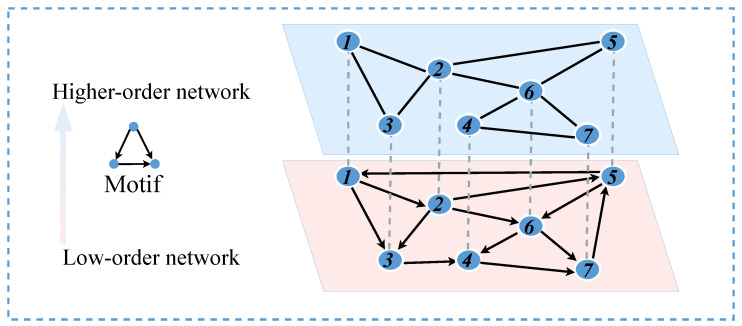
Model of coupled network.

Many studies in the field of complex networks focus on analyzing individual networks. However, many complex networks are coupled and interact in the real world. In a directed network (blue nodes in Figure 3), the theory of higher-order networks proposed by Benson and colleagues is utilized. An adjacency matrix is constructed by counting the occurrences of two nodes appearing together in a motif in the low-order network, which is then used to generate an undirected network representing the corresponding higher-order network (green nodes in Figure 3) [27,28]. When the triangular structures in the higher-order network are disrupted, the corresponding connectivity patterns in the low-order network are also affected. At the same time, the structure of the low-order network also influences the higher-order network. Considering this interactive relationship, the low-order and higher-order networks are coupled, forming a coupled network.

### 3.3. Network Robustness and Network Percolation

If some vertices in a network are removed, along with the edges connected to these vertices, this process is referred to as percolation. When the removed elements are nodes within the network, it is known as site percolation. For instance, in the case of a local area network, when routers experience failures, the corresponding nodes and their interconnecting edges are removed. Similarly, when edges are removed from the network, it is termed bond percolation. For example, in a communication network, communication lines may encounter failures, resulting in the inability of routers to communicate with each other.

**Figure 4:**
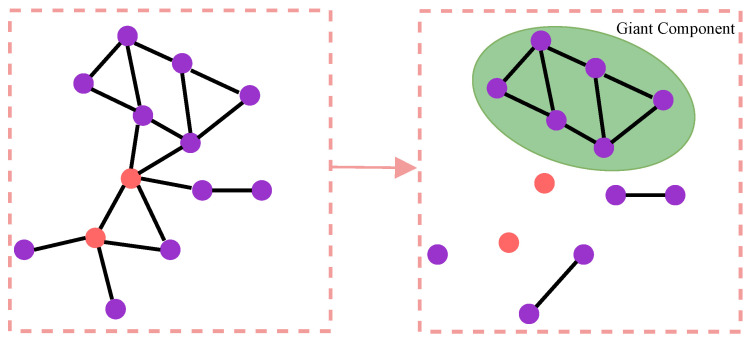
The giant component formed by the remaining nodes after deleting the specified nodes. The nodes highlighted in red in the figure represent the nodes that are to be deleted.

The percolation model is commonly employed to investigate the robustness of complex networks. In this model, a certain proportion of nodes or edges in a network are occupied, and there is subsequent examination of whether the occupied nodes or edges reach the percolation state (whether the occupied nodes or edges can form a network that is comparable to the original network in terms of its functional structure). The size of the giant component formed by the remaining nodes in the network after an attack is an important metric for assessing network robustness. A larger giant component among the remaining nodes indicates stronger network robustness, while a smaller giant component suggests weaker network robustness. As shown in Figure 4, occupying a proportion *p* of nodes in the network is equivalent to deleting a proportion 1−p of nodes from the network. The examination focuses on whether the remaining nodes in the network form a giant component, which is analogous to assessing whether the occupied nodes have reached the percolation state. The formation and disintegration of the giant component are referred to as percolation transition, and the critical value at which the percolation transition occurs is called the percolation threshold. The relative size of the giant component, denoted as P∞, serves as an order parameter.

(1) $$P\infty =\frac{N^{\prime }}{N}$$

Easy to know: P∞∈[0,1]. $N^{\prime }$ represents the number of nodes in the giant component, *N* represents the total number of nodes in the entire network, and *p* is the parameter determining the proportion of remaining nodes after node removal. The critical point $p_{c}$, at which the giant component emerges, is commonly used to measure the robustness of the network. A larger $p_{c}$ indicates poor network robustness; a significant deletion of nodes would cause severe damage to the network. Conversely, a smaller $p_{c}$ indicates good network robustness.

Based on percolation theory, another widely used method to define network robustness relates robustness to the ratio of the largest connected component when nodes are removed. After removing $p^{\prime }$ nodes and summing the proportions of nodes in the largest connected component, denoted as $P\infty (p^{\prime })$, the robustness metric can be represented as follows:

(2) $$R=\frac{1}{N}\sum_{p^{\prime }=1}^{N}P\infty \left(p^{\prime }\right)$$

Here, *N* represents the total number of nodes in the entire network, and $\frac{1}{N}$ is the normalization factor. $R\in [\frac{1}{N},0.5]$; a higher value of *R* indicates stronger network robustness. A lower value of *R* indicates weaker network robustness. In economics, studying the robustness of networks can help identify risks in economic systems [12]. In the case of infrastructure networks, analyzing network robustness enables assessment of the stability of the infrastructure and design of more resilient infrastructure networks [11].

### 3.4. Simulated Annealing Algorithm with Edge Rewiring

Building upon the edge rewiring strategy proposed in Section 4.1, we formulate experiments guided by the principles of simulated annealing. The objective is to enhance the robustness of both high-order and low-order networks concurrently. This approach establishes a higher-order network through motif constructions derived from the lower-order network. Subsequently, two edges are randomly chosen from the lower-order network for edge rewiring, followed by the computation of robustness metrics for both the higher-order and lower-order networks. If the robustness of both networks exhibits simultaneous improvement, the results of the edge rewiring are retained. In cases where the robustness fails to increase concurrently in both networks, there exists a probability of accepting this edge modification. Over a specified number of iterations, the probability parameter (denoted as P) undergoes a gradual reduction (the parameter of simulated annealing can be seen in Section 5). The process of edge rewiring is iterated until a point is reached where the robustness ceases to increase. At this juncture, the algorithm terminates, yielding an optimized lower-order network. The specific algorithmic details and processes are outlined as follows:

Step 1: Randomly select two existing edges, $e_{1}=\left\{v_{i},v_{j}\right\}$ and $e_{2}=\left\{v_{x},v_{y}\right\}$, from the low-order network and rewire them. This results in two new edges, $e_{1}^{\prime }=\left\{v_{i},v_{y}\right\}$ and $e_{2}^{\prime }=\left\{v_{x},v_{j}\right\}$. It is important to note that the new edges should not already exist in the network, ensuring no duplicate edges or self-loops involving the network’s nodes.

Step 2: Calculate the network’s robustness measure *R* after the edge rewiring and use it as the optimization metric. If the robustness of the network is enhanced, then retain the edge rewiring operation. Otherwise, this rewiring process is reserved with a probability (P). Furthermore, the probability (P) decreases as the number of iterations increases.

Step 3: Repeat Steps 1 and 2 until the ratio of effective reconnected edges reaches the required (default is 5%). Stop the iteration to obtain the optimized network.

Where *R* can be obtained by calculating *R* using Equation (2). Define *t* as the improvement rate of robustness; then, we have:

(3) $$t=\frac{R_{after}-R_{before}}{R_{before}}\times 100\%$$

where $R_{before}$ is the initial robustness of the network and $R_{after}$ is the robustness of the network after optimization using the simulated annealing algorithm.

## 4. Network Robustness Optimization Based on Simulated Annealing Algorithm

**Figure 5:**
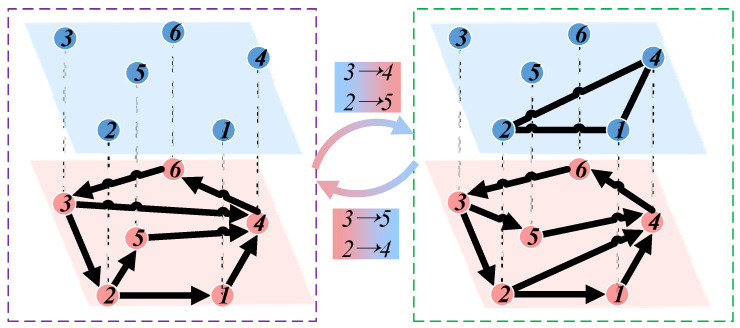
Network Robustness Optimization.

As shown in Figure 5, this section describes the proposed network robustness optimization model based on a simulated annealing algorithm, explicitly focusing on the high-low-order coupled network. The process begins by randomly selecting two edges in the low-order network. These selected edges are then disconnected, and an exchange is made to reconnect them. It is important to note that this process does not alter the node degrees. After the disconnection and reconnection of edges, the robustness of both the low-order and higher-order networks is evaluated. If the robustness of both networks improves, it indicates the effectiveness of the operation, and the reconnected edges are retained in the network. Conversely, if the robustness does not improve, this rewiring process is reserved with a probability (P). During this process, if the ratio of effective reconnected edges reaches the required level, the iteration for reconnecting edges is concluded.

### 4.1. Edge Rewiring

**Figure 6:**
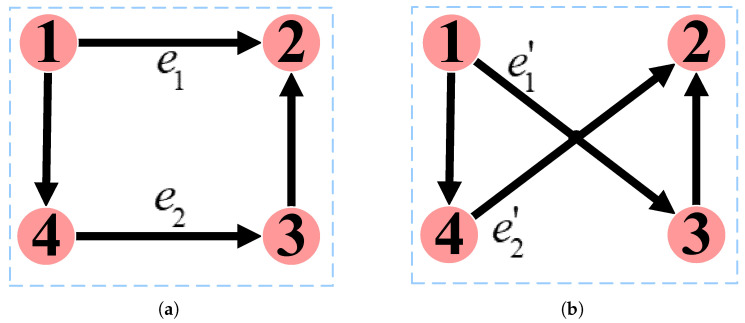
Edge rewiring. (**a**): Before rewiring. (**b**): After rewiring.

Edge rewiring refers to disconnecting edges in a network and then re-establishing the same number of edges according to certain rules. As shown in Figure 6a, the network contains two edges $e_{1}$ and $e_{2}$, where $e_{1}=\left\{v_{1},v_{2}\right\}$, $e_{2}=\left\{v_{4},v_{3}\right\}$. Subsequently, these two edges are disconnected, and a new edge is added between nodes $v_{1}$ and $v_{3}$, and another new edge is added between nodes $v_{4}$ and $v_{2}$. These new edges are denoted $e_{1}^{\prime }=v_{1},v_{3}$ and $e_{2}^{\prime }=v_{4},v_{2}$, respectively. Figure 6b illustrates the resulting network after edge rewiring. It is important to note that this method does not alter the node’s in-degree and out-degree. For instance, the in-degree of node 1 remains 0, while the out-degree remains 2.

### 4.2. Experimental and Evaluation

To study the network’s robustness, we employ malicious node attacks to target the network. Malicious attacks refer to purposefully selecting nodes for targeted attacks. In this study, we employ a high-degree node prioritization attack strategy, which involves first attacking nodes with higher degrees. Malicious attacks expedite the network’s collapse, thereby providing a more intuitive demonstration of the effectiveness of the optimization algorithm. In the second step of the algorithm presented in Section 3.4, three cases need to be discussed:

(1) Optimizing solely based on the robustness of the low-order network.
(2) Optimizing solely based on the robustness of the higher-order network.
(3) Optimizing based on the robustness of both the higher-order and low-order networks.

**Figure 7:**
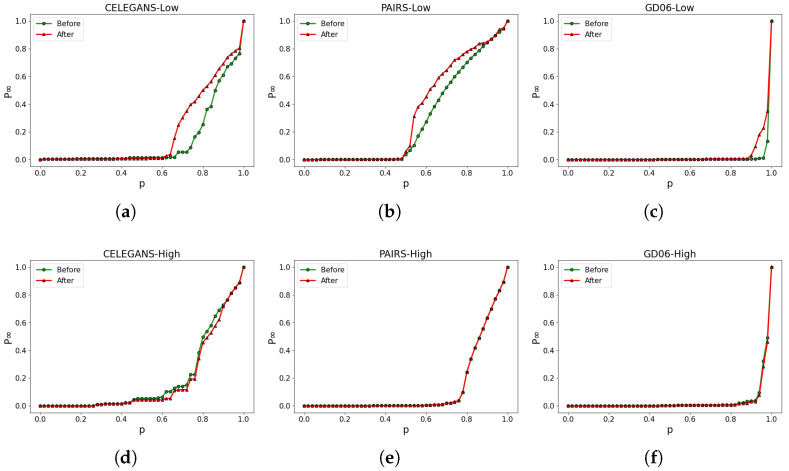
Optimizing based on the robustness of the low-order network only. (**a**–**c**) illustrate the alterations in the robustness of the low-order network, while (**d**–**f**) represent the corresponding modifications in the robustness of the higher-order network.

Our research demonstrates that focusing solely on optimizing the robustness of the low-order network does not necessarily lead to an enhancement in the robustness of the higher-order network. As shown in Figure 7, the experimental results demonstrate that the robustness of the low-order network has been significantly improved, as indicated by the observed enhancements in Figure 7a–c. However, the robustness of the higher-order network shows minimal changes, as depicted in Figure 7d–f. For instance, in Figure 7a, the calculation yields $R_{after}=0.193$, $R_{before}=0.138$, resulting in a value of t=40%. This indicates that the robustness of the low-order network in CELEGANS has been enhanced by approximately 40%. However, in Figure 7d, while the robustness of the low-order network experiences a significant improvement, the robustness of the higher-order network in CELEGANS not only fails to increase but experiences a decrease, from the original $R_{before}=0.138$ to $R_{after}=0.134$. As a result, the overall robustness of the high-low-order coupled network is not enhanced.

**Figure 8:**
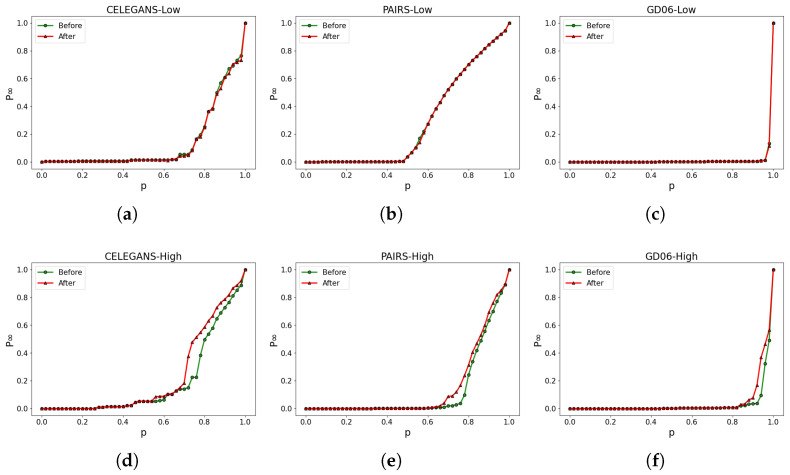
Optimizing solely based on the robustness of the higher-order network. (**a**–**c**) illustrate the alterations in the robustness of the low-order network, while (**d**–**f**) represent the corresponding modifications in the robustness of the higher-order network.

Similarly, optimizing solely based on the robustness of the higher-order network does not necessarily enhance the robustness of the low-order network. As shown in Figure 8, the experimental results reveal that the robustness of the higher-order network exhibits improvements (Figure 8d–f), whereas the robustness of the low-order network displays minimal changes (Figure 8a–c). For instance, in Figure 8d, by computing $R_{after}=0.227$ and $R_{before}=0.192$, we observe a t=18%, indicating an approximate 18% enhancement in the robustness of the higher-order network in CELEGANS. Conversely, in Figure 8a, the robustness of the low-order network showcases negligible variations, maintaining results comparable to the initial state. Consequently, the overall optimization of the high-low-order coupled network’s robustness has not been effectively achieved.

In summary, improving the high-low-order coupled network’s robustness necessitates considering both the low-order and higher-order networks’ robustness. Consequently, the subsequent approach detailed in this paper considers both networks’ robustness as optimization criteria, utilizing a simulated annealing algorithm to optimize the robustness of the high-low-order coupled network effectively.

## 5. Experimental Simulation and Analysis

The simulated annealing algorithm we designed needs to have an initial probability of P and the value of P gradually decreases in the process. P determines the probability of accepting the edge rewiring even if the robustness of the network decreases after the edge rewiring. If P is too small, the algorithm tends to be a greedy algorithm, and in the case of a large value of P, the algorithm tends to be stochastic. Therefore, P is a very important parameter; according to our experiment, we choose P = 0.01 and set P = P − 0.001 after every 1000 edge rewiring. Our experiment showed that the algorithm performs well under the parameters.

### 5.1. Robustness Optimization of Coupled Networks Based on Three Classic Networks

#### 5.1.1. Data Description

In this study, three well-known undirected networks are employed: the Erdős–Rényi (ER) network [29], the Barabási–Albert (BA) network [30,31], and the small-world (SW) network [32]. Each network has an average degree of <k>. Subsequently, each undirected edge was assigned a random direction to introduce directionality, thereby transforming these three undirected networks into directed networks. The references to ER, BA, and SW networks in the subsequent text pertain to their corresponding directed networks. Given that real-world networks typically have a relatively low number of bidirectional edges, this study does not consider bidirectional edges. For instance, in the neural network CELEGANS, the proportion of bidirectional edges is merely 8.4% [32], while in the chess competition network CHESS, it is only 6.9% [33]. If not explicitly stated, the default number of nodes in the generated networks is 1000.

#### 5.1.2. Results Analysis

**Figure 9:**
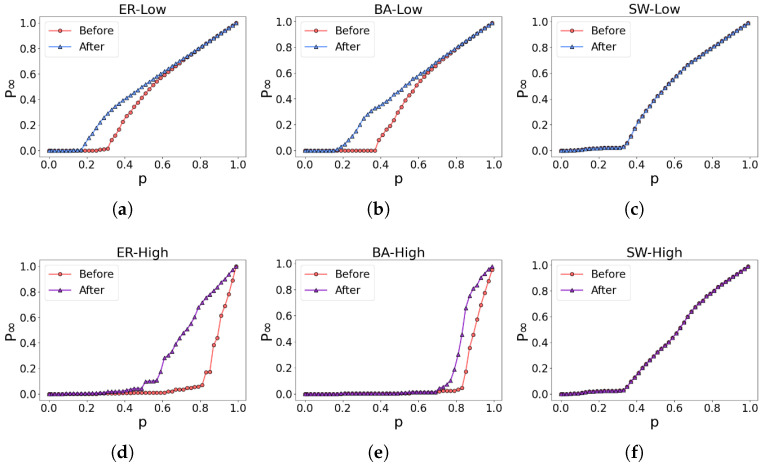
Robustness optimization of three classic networks. (**a**–**c**) illustrate the alterations in the robustness of the low-order network, while (**d**–**f**) represent the corresponding modifications in the robustness of the higher-order network.

The experimental results are depicted in Figure 9. In the ER network and BA network, both the low-order and higher-order networks demonstrate improved robustness, as evident in Figure 9a,b,d,e. However, in the case of the SW network, the network’s robustness remains relatively unchanged, as illustrated in Figure 9c,f.

**Figure 10:**
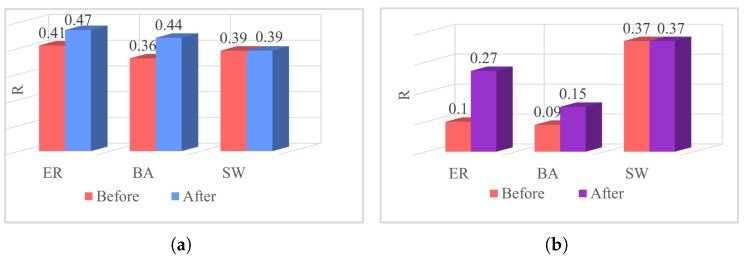
Robustness optimization of three classic networks. (**a**): Robustness of low-order networks. (**b**): Robustness of higher-order networks.

We examined the factors contributing to the unaltered robustness of the SW network by evaluating the parameter *R*. As depicted in Figure 10, Figure 10a,b illustrate the optimization of robustness in the low-order and higher-order networks, respectively. It was observed that the higher-order network demonstrates robustness from the outset within the SW network. For example, the initial robustness of the ER network’s higher-order network is 0.1, while in the SW network, it is 0.37–3.7 times higher than that of the ER network. Thus, when evaluating robustness optimization criteria for both low-order and higher-order networks, no further enhancement is feasible for the higher-order network in the SW network. Consequently, neither the low-order nor the higher-order networks in the SW network attain optimized robustness, underscoring the intrinsic robustness of the SW network.

Table 1 illustrates the results obtained from optimizing the robustness of the three aforementioned classical networks mentioned above. By strategically exchanging a proportion of $e_{e}$ edges within the network, we observed the resulting changes in network robustness. The improvement rates of robustness for the low-order and higher-order networks are denoted by $t_{low}$ and $t_{high}$, respectively. The initial portion of the ± signs represents the percentage of robustness improvement, while the latter half denotes the standard deviation of the data. For the ER and BA networks, both the low-order and higher-order networks manifested specific improvements in robustness. As an example, the robustness of the higher-order network in the ER network increased by 162%, whereas the low-order network’s robustness improved by 14.6%. The relatively modest enhancement in the robustness of the low-order network can be attributed to its already strong robustness at the outset. Consequently, despite optimization endeavors, the degree of enhancement remains constrained.

The experiments demonstrate that network robustness can be enhanced through effective edge rewiring. Additionally, as presented in Table 2, Table 3 and Table 4, we collected data on network characteristics before and after optimization for the three networks. In the low-order network, the optimized ER and BA networks exhibited enhancements in the average shortest path length <d>, the average clustering coefficient *C*, and the degree assortativity *r*.

### 5.2. Robustness Optimization of Coupled Networks Based on Real-World Networks

The preceding section successfully showcased the efficacy of the proposed robustness optimization algorithm through experiments conducted on three classical networks. In the subsequent section, the study aims to further evaluate the algorithm’s effectiveness by subjecting it to testing on 14 real-world networks.

#### 5.2.1. Data Description

The experimental datasets consist of 14 directed networks from diverse domains, including CELEGANS [32]; EMAIL, GD06, TRUST, SPAM, PAIRS, PAGES, CHESS, CORA [33]; POLBLOGS [34]; CL-1000, MARAGAL, UTM3060 [35]; and ODLIS [36]. A brief description of each dataset is as follows:

(1) CELEGANS: A neural network of the nematode worm, where edges represent synaptic or gap junction connections between neurons.
(2) EMAIL: A directed email communication network where nodes correspond to users and directed edges represent email exchanges between users.
(3) GD06: A software class dependency network where nodes represent classes and edges indicate dependencies between classes.
(4) TRUST: A user trust network where nodes represent users and edges represent trust relationships between users.
(5) SPAM: A hyperlink network indicating pages pointing to other pages.
(6) PAIRS: A network where nodes represent words and edges represent related words associated with a particular word.
(7) PAGES: A social network representing user-following relationships.
(8) CHESS: A network of international chess competitions where nodes represent players and edges represent matches between players.
(9) CORA: A citation network of scientific papers where nodes represent papers and edges represent paper-to-paper citations.
(10) POLBLOGS: A hyperlink network among U.S. political blogs.
(11) CL-1000, UTM3060, and MARAGAL: Networks obtained from Internet downloads.
(12) ODLIS: An online dictionary network where nodes represent terms and edges represent one term describing the meaning of another term.

In Section 3.1 and Section 3.2, we expounded on the genesis of high-order networks and underscored the significance of coupled high-order and low-order networks. Scholars have validated this theoretical framework in diverse domains, such as social, Internet, biological, and information networks. To better substantiate our experimental findings, the network datasets are carefully selected to align with these conditions. For instance, datasets like EMAIL, TRUST, PAGES, POLBLOGS, and CHESS fall under the category of social networks. Similar considerations apply to the other network types.

The structural statistical features of these experimental datasets are presented in Table 5, arranged in ascending order based on node count. It is crucial to emphasize that, due to the lack of strong connectivity in certain networks, experiments were performed using the largest strongly connected component of each network [10].

#### 5.2.2. Results Analysis

**Figure 11:**
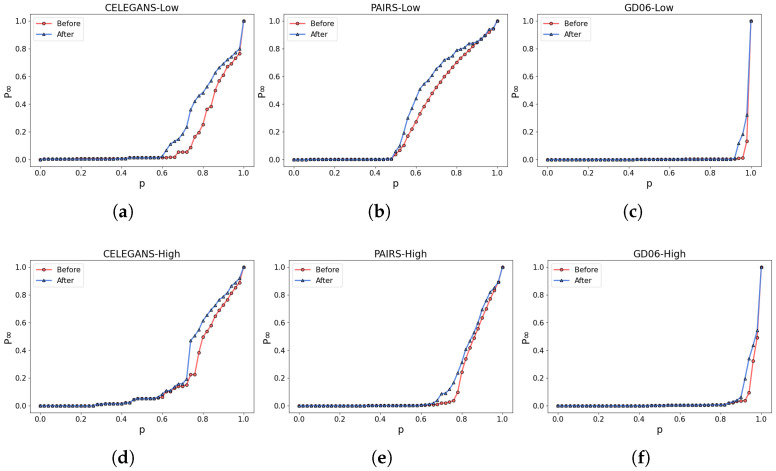
Network Robustness Optimization In Real-World Networks. (**a**–**c**) illustrate the alterations in the robustness of the low-order network, while (**d**–**f**) represent the corresponding modifications in the robustness of the higher-order network.

The partial visualization results of the experiments are shown in Figure 11a,b. By incorporating the robustness of both the low-order and higher-order networks as optimization criteria, the ratio of edge rewiring is controlled at 5%. As a result, CELEGANS, PAIRS, and GD06 exhibit specific improvements in low-order and high-order networks.

**Figure 12:**
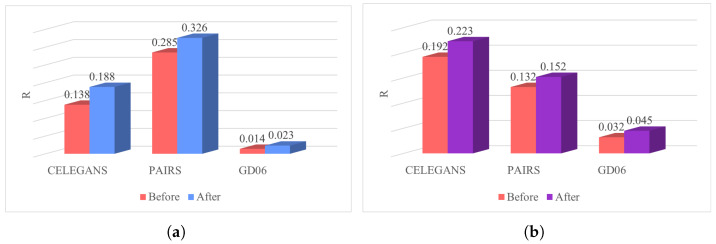
Robustness optimization of three classic networks. (**a**): Robustness of low-order networks. (**b**): Robustness of higher-order networks.

To visually assess the robustness of the networks before and after optimization, Figure 12 is presented, which compares the differences in robustness by calculating the *R* values. The robustness of the networks significantly improves through the optimization process employing the simulated annealing algorithm. For instance, in the CELEGANS network, the robustness of the low-order network before optimization is $R_{before}=0.138$, and after optimization, it is $R_{after}=0.188$. This signifies an enhancement of 0.15 in the robustness of the low-order network, corresponding to a percentage increase of 36%(t=36%). Likewise, the percentage increase in robustness for the higher-order network is calculated as 16%. The optimized networks demonstrate a noteworthy enhancement in robustness.

The experimental results for the 14 real-world networks after optimization are summarized in Table 6. To minimize the error due to randomness, we averaged the results of all experiments 10 times. As mentioned in the previous section, the initial portion of the ± signs represents the percentage of robustness improvement, while the latter half denotes the standard deviation of the data. Through the effective 5% edge swapped strategy, the robustness of all networks has been significantly improved. The data clearly demonstrate that even by optimizing a small fraction of edges in the low-order network, both the low-order and higher-order networks can experience substantial enhancements in robustness, leading to an overall improvement in the robustness of the high-low-order coupled network. As an example, in the GD06 network, with only 5% of edges swapped, the robustness of the low-order network increased by 64.7%, while the robustness of the higher-order network increased by 37.9%. This optimization effect is noteworthy. When both the low-order and higher-order networks exhibit improved robustness, it naturally translates to a heightened overall robustness of the high-low-order coupled network.

## 6. Conclusions

This paper proposes a simulated annealing optimization algorithm to enhance the robustness of high-low-order coupled directed networks. The proposed method simultaneously considers the robustness of both the low-order and higher-order networks as optimization objectives. Unlike traditional methods that focus solely on improving the robustness of the low-order network, the proposed algorithm optimizes the robustness of both the low-order and higher-order networks. By rewiring a small fraction of edges in low-order networks, while preserving the degree distribution of network nodes, the algorithm achieves improved robustness in low-order and higher-order networks.

The effectiveness of the proposed method is demonstrated through an experimental analysis conducted on ER, BA, and SW networks, as well as 14 real-world networks. The results show that the algorithm effectively improves the robustness of both low-order and higher-order networks. When both networks exhibit improved robustness, the overall robustness of the high-low-order coupled network is also enhanced. Notably, significant enhancements in robustness are achieved by optimizing only 5% of the edges in the networks. Additionally, from a holistic perspective, as the number of network nodes increases, the computational requirements of our algorithm demonstrate nearly linear growth.

It is important to note that the proposed method is tailored explicitly for directed networks, and its applicability to undirected networks necessitates additional investigation. The approach based on the simulated annealing algorithm may also encounter limitations, such as slow convergence speed and inability to guarantee the global optimal solution. Potential future research directions could explore alternative methods, including heuristic approaches, methods based on complex network dynamics, or deep-learning-based methods. These avenues aim to further enhance the robustness of coupled high-low-order networks and overcome the limitations of the simulated annealing algorithm.

## Figures and Tables

**Table 1:** Robustness optimization of three classic networks.

Network	$e_{e}$	$t_{\mathrm{low}}$	$t_{\mathrm{high}}$
ER	5%	14.6% ± 0.23%	162% ± 4.15%
BA	5%	20.9% ± 0.82%	64.5% ± 1.02%
SW	0%	0%	0%

**Table 2:** Statistical properties of the ER network.

Network	N	M	<k>	<d>	C	r
Low-order Network	1000	8000	16	3.554	0.008	0.008
Optimized Low-order Network	1000	8000	16	3.563	0.011	0.032
Higher-order Network	1000	1348	2.696	6.460	0.602	−0.038
Optimized Higher-order Network	1000	1539	3.078	5.594	0.496	−0.069

**Table 3:** Statistical properties of the BA network.

Network	N	M	<k>	<d>	C	r
Low-order Network	1000	8000	16	3.554	0.012	0.260
Optimized Low-order Network	1000	8000	16	3.565	0.014	0.276
Higher-order Network	1000	2341	4.682	3.697	0.506	0.286
Optimized Higher-order Network	1000	2631	5.262	3.833	0.435	0.367

**Table 4:** Statistical properties of the SW network. Due to the inherent robustness of the SW network, the network did not achieve significant improvement through optimization. ∼ indicates that the corresponding values remained unchanged after optimization.

Network	N	M	<k>	<d>	C	r
Low-order Network	1000	8000	16	4.688	0.263	0.001
Optimized Low-order Network	∼	∼	∼	∼	∼	∼
Higher-order Network	1000	7276	14.552	20.128	0.632	−0.008
Optimized Higher-order Network	∼	∼	∼	∼	∼	∼

**Table 5:** Statistical characteristics of real-world networks. Note: *N* represents the number of nodes, *M* represents the number of edges, <k> represents the average degree of the network, <d> represents the average shortest path length, *C* represents the clustering coefficient, *r* represents the degree assortativity.

Network	*N*	*M*	<k>	<d>	C	r
CELEGANS	297	2345	15.79	3.99	0.17	−0.26
EMAIL	906	12,085	26.68	2.68	0.34	0.08
CL-1000	928	4897	10.55	3.26	0.10	−0.07
POLBLOGS	1224	19,022	31.08	3.19	0.22	−0.19
GD06	1538	8032	10.44	5.21	0.22	−0.12
MARAGAL	1964	26,692	27.18	3.23	0.10	−0.14
ODLIS	2900	18,241	12.58	4.59	0.18	0.01
UTM3060	3060	39,151	25.59	14.43	0.39	0.34
TRUST	4658	40,133	17.23	2.90	0.09	0.11
SPAM	4767	37,375	15.68	3.81	0.14	0.04
PAIRS	5018	63,608	25.35	4.26	0.13	−0.02
PAGES	7057	89,429	25.34	4.25	0.21	0.07
CHESS	7301	60,046	16.45	4.29	0.10	0.39
CORA	23,166	91,500	7.90	13.33	0.15	0.02

**Table 6:** Enhancement of network robustness. $e_{e}$ represents the percentage of edges effectively swapped in the low-order network. $t_{low}$ denotes the improvement in robustness of the low-order network and $t_{high}$ represents the improvement in robustness of the higher-order network.

Network	$e_{e}$	$t_{\mathrm{low}}$	$t_{\mathrm{high}}$
CELEGANS	5%	36.1% ± 0.44%	16.1% ± 0.75%
EMAIL	5%	41.1% ± 0.32%	34.3% ± 0.42%
CL-1000	5%	21.2% ± 0.04%	22.1% ± 0.04%
POLBLOGS	5%	60.1% ± 0.92%	53.4% ± 1.18%
GD06	5%	64.7% ± 0.49%	37.9% ± 0.51%
MARAGAL	5%	18.2% ± 0.03%	22.3% ± 0.07%
ODLIS	5%	59.5% ± 0.62%	31.1% ± 0.39%
UTM3060	5%	30.7% ± 0.37%	29.1% ± 0.33%
TRUST	5%	42.3% ± 0.71%	33.5% ± 0.66%
SPAM	5%	69.3% ± 1.24%	75.2% ± 1.12%
PAIRS	5%	14.9% ± 0.02%	15.3% ± 0.04%
PAGES	5%	19.4% ± 0.13%	17.1% ± 0.52%
CHESS	5%	65.2% ± 1.78%	58.9% ± 1.32%
CORA	5%	45.1% ± 0.79%	39.4% ± 0.83%

## Data Availability

Data available on request from the authors.
